# Women’s experience of gestational diabetes and healthcare in southern Sweden – a qualitative study

**DOI:** 10.1186/s12884-025-07328-2

**Published:** 2025-02-28

**Authors:** Amanda Björk Javanshiri, Sara Modig, Peter Nymberg, Susanna Calling

**Affiliations:** 1 Center for Primary Health Care Research, Department of Clinical Sciences Malmö, Lund University, Malmö, Sweden; 2 Office for Primary Care, Skåne University Hospital, Lund, Sweden

**Keywords:** Gestational diabetes mellitus, Qualitative research, Semi-structured interviews, Pregnancy care, Care experience, Patient experience, Healthcare organization

## Abstract

**Background:**

Gestational diabetes is one of the most common pregnancy complications, affecting 14% of all pregnancies globally, and its prevalence is increasing. Gestational diabetes is associated with short and long-term complications for both the mother and their offspring, which are possible to prevent by glycemic control mainly facilitated by healthy lifestyle behaviors. Hence, women diagnosed with gestational diabetes have a significant role in disease management which can be perceived as burdensome. Previous research has well documented the psychological implications of diagnosis in the women and the need for support from healthcare. Despite the global burden of disease associated with gestational diabetes, recent qualitative studies exploring women's experiences are scarce, particularly in Sweden. Thus, highlighting a critical gap in understanding the impact of this condition and the women's experiences of diagnosis and prenatal healthcare, which this study aimed to address.

**Methods:**

Purposive sampling was used to recruit women with previous gestational diabetes in southern Sweden. Individual interviews were held with 17 participants according to a semi-structured interview guide. The interviews were audio recorded and transcribed verbatim. Data analysis was conducted according to qualitative content analysis.

**Results:**

The analysis generated 10 subcategories, which were grouped into three categories labeled: “experience of diagnosis”, “a complex relationship with food” and “experience of prenatal healthcare”. One theme emerged: to suddenly become a patient as opposed to an expectant mother. Most women were initially overwhelmed by the diagnosis and its consequences. They felt that healthy eating was important, despite it limiting their everyday lives, which also revealed a complicated relationship with food. Women felt supported during pregnancy but abandoned after labor. They requested additional information and emotional support from healthcare personnel, increased awareness and understanding of the treatment burden of gestational diabetes, improved person-centered care, and collaboration between healthcare providers, particularly to ensure better support in reducing future risk of disease.

**Conclusions:**

This study provides an understanding of women’s experience of gestational diabetes and the care provided in southern Sweden. Their views could improve future care regarding both successful gestational diabetes management and post-pregnancy follow-up to prevent long-term adverse health outcomes.

## Background

Gestational diabetes mellitus (GDM), high blood glucose first recognized during pregnancy, is one of the most common pregnancy complications. Its prevalence is increasing globally, affecting 14% of all pregnancies [1]. According to the Swedish Pregnancy Register, the incidence of GDM in Sweden in 2022 was 6.5% [2]. The condition is usually discovered by screening with an oral glucose tolerance test (OGTT) [3, 4]. GDM increases the risk of complications during pregnancy and labor, including but not limited to; preeclampsia, macrosomia, shoulder dystocia and caesarean section [5]. After delivery most women recover from the condition, hence GDM can be regarded as a transient state. However, GDM also has significant implications for women’s long-term health since it is considered a major risk factor for future type-2 diabetes mellitus (T2DM) and cardiovascular disease as well as increased mortality [6–9]. GDM also increases the risk of obesity, cardiovascular disease, and T2DM in the women’s offspring [10–13]. Glycemic control is important to minimize the risk of adverse maternal, fetal, and long-term outcomes [5, 14]. This can be obtained by preventive lifestyle interventions, a healthy diet, and regular exercise both during and after pregnancy [14, 15]. Throughout pregnancy, GDM is managed by dietary modifications based on self-monitoring and reporting of blood glucose levels as well as the use of glucose-lowering treatment if needed, which 25% of women with GDM require [15]. Women in southern Sweden with GDM are followed by a multidisciplinary team consisting of midwives who conduct prenatal controls, extra scans to monitor fetal growth, dietary advice by a dietitian, weekly contact with endocrinologists, and birth planning with an obstetrician [16]. However, this also means a shift in normal pregnancy, with increased surveillance and the risk of women’s pregnancies becoming medicalized [17].

Consequently, GDM has a major impact on women’s lives, pregnancies, and future health for themselves as well as their children. Earlier research has described the experience of GDM as a process from “stun to gradual balance” [18]. Multiple qualitative studies have revealed the psychological impact following a GDM diagnosis on women and the importance of sufficient support from healthcare personnel [19–22]. Women’s attitudes might also influence how responsive they are to interventions both during and after pregnancy. We believe that for successful GDM management, it is essential to incorporate the perspectives of the patient group. Thus, there is a need to investigate women’s experiences of the diagnosis and prenatal healthcare. To our current knowledge, there have not been any recent qualitative studies on women with GDM in Sweden, which this study provides novel insight into.

## Methods

### Aim, design and setting

This study aimed to explore women with a recent history of GDM in southern Sweden and their experience of the GDM diagnosis and prenatal healthcare. A qualitative approach was found most suitable to answer the research question. The study was conducted in primary care in southern Sweden, Skåne county. It follows the Consolidated Criteria for Reporting Qualitative research (COREQ) [23].

### Data sources and sampling

The study population data were retrieved from women diagnosed with GDM, ICD O24.4 and O24.4X (International Classification of Diseases, ICD-10) at the Endocrinology Department, Skåne University Hospital in Malmö and Lund during 2021- 2022 (N = 784). The diagnosis was then linked to a personal identification number. This data sampling method was chosen because women diagnosed with GDM in southern Sweden are referred to the specialist prenatal care unit as well as the endocrinology department for follow-up during pregnancy. Eligible women were invited by postal mail (approximately 20 at a time), with information about the study, offering to participate in face-to-face interviews according to a semi-structured interview guide at a dedicated primary health care center. If difficulty participating in face-to-face interviews was encountered other interviewing alternatives digitally via Zoom video platform or by phone were offered to facilitate recruitment. Purposive sampling was used to obtain maximum variation, to include participants of different ages, parity, socioeconomic status, habitation, and ethnicity.

The following participants were excluded:

- Women diagnosed with recurrent GDM, type 1 or 2 diabetes.

- Women who had moved from the geographically relevant area.

- Women who could not communicate in Swedish.

A semi-structured interview guide with open-ended questions was developed based on different thematic areas of interest by three co-authors. Two pilot interviews, which are included in the analysis, were conducted to test for relevance, resulting in minor adjustments of the interview guide. The interview guide ended with a question exploring whether the participants would like to add anything.

The following thematic areas were explored:

- Overall experience of GDM and perceived risk of developing T2DM.
- Overall experience of the care provided as well as barriers and facilitators for attending regular follow-up during pregnancy.

Two weeks after the invitations were sent, the women were contacted by phone for further information and to answer potential questions. If a woman wished to take part in the interviews, written or verbal consent was collected before the interviews. All interviews were conducted in Swedish, between September and December 2023 according to the semi-structured interview guide but with the possibility to explore other themes presented with probing questions during the conversation. Nine participants preferred face-to-face interviews, seven favored phone interviews and one chose to be interviewed via Zoom video platform (® version 5.17.11). The individual interviews took place at a dedicated primary healthcare center (the first author’s workplace) in a neutral room. Participants were offered water, tea or coffee but received no economic compensation for participating. All interviews ended with an open question about whether the participants had anything to add, as well as simple questions about demographics, age, number of children, weight and height, smoking, country of origin and education. Field notes were taken during the audio recorded interviews, which were then transcribed pseudonymized. Interviews were conducted until further interviews did not render any more information, after 17 interviews when data saturation were deemed to have been reached.

### Analyses

The data analysis was conducted according to a qualitative content analysis approach as described by Graneheim and Lundman [24]. Transcribed interviews were read several times through to get a sense of the whole. For each interview, meaning-bearing units were identified, condensed, and labeled with a code by the first author in consultation with the other authors. These codes were used to synthesize all interviews into subcategories that were inductively grouped into categories that were discussed and interpreted by each involved co-author until consensus was reached. We used a native speaking science editor to revise the manuscript with special attention to the English translation of quotations, subcategories and categories. To ensure the integrity of the content, we translated the citations both forward and backward. This process was thoroughly conducted to maintain the original meaning and accuracy of the information. Examples of the analysis from meaning-bearing units to categories are shown in Table 1.

**Table 1:** Example of text condensation and coding

Meaning unit	Condensed meaning unit	Code	Subcategory	Category
*When you were told that…that it could affect the child and that the baby can grow quite a lot bigger, and then you just felt, but I don’t want that, that my child will grow extra-large. Because I’ve gone…like maybe you’ve eaten unhealthily or haven’t thought about what you put in your mouth* (Participant 11)	Didn’t want the child to grow too large, because of what I ate	To be responsible, managing GDM by diet	Consequences	Experience of diagnosis
*Even if you’re not there anymore, it is not visible in that way. But that doesn’t mean that you still can’t have those thought patterns. Even if you don’t look thin as a rail, that doesn’t mean that you still don’t have it mentally. And I guess that I didn’t feel that awareness from healthcare at that time.* (Participant 10)	Even though you aren’t thin as a rail, you can still have those thoughts, which healthcare should be aware of	Attention to underlying eating disorder	Underlying eating disorder	A complex relationship with food
*Yes, but I had her for over ten years. So, she had been with me since the first contraceptive and basically everything. // So, I didn’t want to leave her and go to specialist maternity care that don’t know me at all.* (Participant 9)	Didn’t want to leave the midwife who knew me and go to the specialist prenatal care who didn’t	Wanting to feel safe and trust healthcare	Person-centered care	Experience of prenatal healthcare

### Researcher characteristics, preunderstanding, and reflexivity

The subject of this qualitative study was selected by the researchers involved since we perceived this as an uncharted area in need of investigation. Data collection, invitations, and interviews, as well as data analysis, were performed by the first author (ABJ), a female M.D and PhD student doing her residency in general practice also working at a midwifery clinic. Normally, GDM care is not provided in Swedish primary care during pregnancy, but primary care is responsible for the postpartum follow-up. In southern Sweden, women with GDM are managed by a multidisciplinary team at the hospital during pregnancy. Since ABJ also is involved in reviewing local guidelines on follow-up of women with previous GDM in primary care, she collaborated with both obstetricians and endocrinologists in the field, and these professionals provided important knowledge but weren’t involved with the study as such. The senior authors (SC and SM), both female associate professors and general practitioners with experience in conducting qualitative research provided guidance and participated in the data analysis. The remaining senior author (PN) later joined as an external reviewer of the interview guide and participated fully in the data analysis, a male general practice registered nurse with a PhD and qualitative research experience. Hence, all authors were employed in primary care but had no medical responsibility for the included participants.

## Results

A total of 62 women were invited; half of the invited (n = 31) did not answer when contacted by phone, eight declined participation, two were diagnosed with recurrent GDM/T2DM, two were misdiagnosed (meaning that one did not suffer from GDM and the other was diagnosed with type 1 diabetes) and two agreed to participate but did not attend their scheduled interviews and were unreachable thereafter. Altoghether, 17 women registered to different primary healthcare centers consented to participate. The interviews lasted between 18 min and 1 h and 16 min and were, on average 37 min. Characteristics of the study population are listed in Table 2. The median age was 32 years, and the participants ranged from 26 to 43 years. They had one or two children and a median body mass index (BMI) of 30,1 (two participants did not want to state their weight). Three had other countries of origin than Sweden. All were non-smokers, and the majority had higher education (college or bachelor’s degree).

**Table 2:** Characteristics of study population (*n* = 17)

Characteristic		Median (range)	N (%)
Age, years		32 (26–43)	
No. of children		1 (1–2)	
Body mass index (BMI)		30,1 (21.2–37.8)	
Smoking	Never smoked		12 (71)
	Former smoker		5 (29)
	Smoker		0 (0)
Ethnic background	Sweden		14 (82)
	European		1 (5)
	Non-European		2 (12)
Education	Elementary school		1 (5)
	Secondary school		4 (24)
	University		12 (71)

**Fig. 1:**
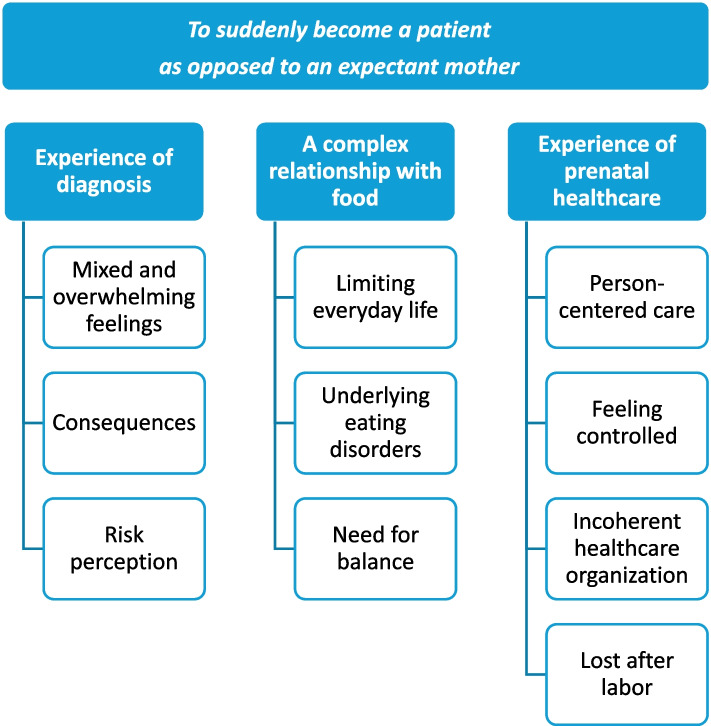
Overview of subcategories, categories and theme

The analysis resulted in 10 subcategories, grouped into three categories: experience of diagnosis, a complex relationship with food and experience of prenatal healthcare, illustrated in Fig. 1. One theme emerged: to suddenly become a patient as opposed to an expectant mother.

Listed below are the categories with subcategories in *italics* embedded within the text, some illustrated with direct quotes.

### Experience of diagnosis

This category covered mixed and overwhelming feelings, consequences, and risk perception.

Women diagnosed with GDM experienced *mixed and overwhelming feelings*. The majority reported feelings of shock, sadness, disappointment, and powerlessness when informed. Many did not consider themselves as being at risk, aware of GDM being associated with heredity, unhealthy lifestyles, and obesity, and therefore felt surprised.

“*I was very surprised, because no one else in the family has experienced this, and then I felt why me, in that moment.”* (Participant 11).

Even those aware of the increased risk of GDM felt surprised. A few thought that it was somewhat expected because of a family history of diabetes, former GDM, or being overweight. Others felt that they knew very little about the condition. Many expressed thoughts about GDM being self-inflicted and felt guilt, shame as well as embarrassment because of it.

“*Yes, but a little embarrassing, you don’t want to…I’m quite ashamed that I’ve gained so much weight and then I’m getting a disease because of it, which can also affect my child, so then it feels a little irresponsible, like, almost a little ashamed.*” (Participant 15).

Feelings of failure and grief that GDM disrupted their pregnancies were also present. Some described stigmatization as being labeled by both society and healthcare personnel, and a feeling of being judged by assumptions about influenceable risk factors.

“*That you end up in such a subcategory of pregnancy. That you are not a perfect pregnant woman*” (Participant 10).

This was reinforced by women with GDM being followed in a specialist diabetes/prenatal setting at the hospital rather than the ordinary maternity clinic. A few women also felt disbelief and doubted whether they had GDM since they did not experience any symptoms of the condition. They struggled to come to terms with the diagnosis and expressed suspiciousness about the screening method and cut-off values for GDM since their self-monitoring glucose levels were often normal. The initial overwhelming feelings were followed by uncertainty and fear not knowing what GDM would imply for the women themselves or for their expected child. These feelings were strengthened by the fact that many felt insufficiently informed by healthcare personnel when diagnosed. Most women gradually experienced acceptance and found a way to handle GDM in their everyday lives. Acceptance was facilitated by GDM being a temporary, transitory state and the health of their unborn child being their main priority. A sense of liberation and relief was evidently reported after childbirth, in stark contrast to the initial shock when diagnosed.

“*I was mostly just relieved that it was done, that it was over. And felt like this; now I’m rid of it, it felt like*” (Participant 10).

Overall, participants reported that GDM had several *consequences* related to treatment burden, medicalization of pregnancy, loss of control vs body awareness, and fetal risks. Many described the treatment burden of GDM; as frequently self-monitoring blood glucose several times a day, attending extra prenatal controls as well as ultrasounds, and having regular contact with the hospital during pregnancy was experienced as strained and time-consuming. Overall, it was perceived that this limited their daily lives. Some even expressed that they were “lucky enough” to be unemployed during their pregnancies since GDM occupied all their time. This also contributed to a medicalization of their pregnancies, as some women felt that their pregnancy was more about GDM than being pregnant.

“*So it became very much like my whole pregnancy revolved around that, instead of revolving around the arrival of a child*.” (Participant 10).

The fact that GDM usually does not cause any symptoms gave some participants a feeling of loss of control. This was sometimes balanced by regular self-monitoring of blood glucose, which gave some women a sense of body awareness and stress relief. Many women expressed concern that GDM or treatment would affect the health and well-being of their unborn baby both short- and long-term, worrying about macrosomia, induced delivery, and future diabetes risk in the child. In a sense, some also felt responsible for this as expectant mothers.

“*I thought some about the fact that it could affect my child, that I had to think about what I ate and how I ate, so that she wouldn’t be affected*.” (Participant 6).

A few women had babies that were small for gestational age and perceived that this was due to strictly following dietarian advice. One participant reported that GDM meant that she was encouraged by some healthcare personnel to give her baby formula after delivery due to the risk of infant hypoglycemia, despite her desire to only breastfeed.

Most had an increased *risk perception* and perceived that they themselves were at greater risk of future diabetes. All participants were informed of the increased risk of T2DM. They were also aware of other risk factors such as heredity, obesity, and unhealthy lifestyles (diet and physical activity), with the latter two being seen as modifiable risk factors that could help reduce their risk. Many expressed great concern about recurrent GDM, that they would not recover from the condition after labor or that it would develop into T2DM. They feared having to face living with a chronic illness. They also reported that regular follow-up postpartum would decrease this concern. On the other hand, some said that they were aware of the increased risk of later T2DM but were not worried about it, that it was not a daily concern, rather that it was in the back of their minds.

“*So yes, the experience is that it is in the back, that is, in the back of my mind. You think about it a little bit, but not that much*.” (Participant 5).

Some participants described that their experience of GDM made them less concerned about future diabetes because of knowledge about how to manage it.

“*But then I also think, it wasn’t the end of the world. It still went pretty well*.” (Participant 2).

A few expressed a wish to forget about it all, describing how they put the blood glucose monitor in a cabinet to forget about GDM, both literally and figuratively. Only one participant neglected her own risk of future T2DM. Despite being aware of the risk association, she didn’t believe that she would ever suffer from T2DM in the future.

### A complex relationship with food

For many women, GDM entailed persistent healthy eating to gain glycemic control and revealed a complex relationship with food involving limiting everyday life, underlying eating disorders, and a need for balance.

Some believed that they already ate healthily, which perhaps also contributed to their surprise at the diagnosis. Managing the disease with a healthy diet was perceived by many as *limiting their everyday life* and affecting their quality of life. Women reported having to think about what they ate constantly, making it difficult to enjoy both life and the pregnancy. Some felt that food and mood were connected, feeling sad and restricted by dietary advice.

“*It almost felt like you were on a diet, because you didn’t want the blood glucose to get too high, but it felt like whatever I did, and even though I pushed myself it wasn’t enough*” (Participant 11).

Several described that this, combined with regular monitoring and reporting their blood glucose levels, could trigger a previous, or potentially induce, *eating disorder*. For instance, thoughts of using Metformin for weight loss after pregnancy were expressed. It was moreover implied that healthcare staff did not have sufficient awareness or knowledge of this, failing to offer enough emotional support. A few also reported that these thoughts and behaviors continued to some extent postpartum.

“*You got a bit of disturbed behavior and thoughts from controlling your diet and blood glucose so closely, for such a long time…//and I’ve never, perhaps I should add, had any problems with eating disorders or anything like that. But my feeling was that oh, if you had, this would’ve been difficult. Having to evaluate the food you ate so much. And those thoughts, I feel, have stuck with me*.” (Participant 17).

To successfully manage the condition with diet alone instead of insulin was perceived by many as a success and relief. As previously stated, many women expressed that they felt limited by strictly healthy eating during pregnancy and that this also was a concern if they in the future would suffer from T2DM. Most participants reported that they continued with healthy eating to some extent postpartum but that it was difficult to sustain, and some relapsed into unhealthy eating habits. They expressed a *need for balance*, to “treat” themselves with food, which sometimes felt important for their, and their partner’s, well-being. Some described unhealthy eating during evenings with their partner after putting their children to bed as a guilty pleasure. Others described food for comfort and emotional eating as a way of controlling feelings. However, permitting oneself to indulge sometimes also made it easier to eat healthily the rest of the time.

“*I feel now that I don’t exclude anything that is, that I can sometimes eat a hamburger or pizza, or some candy on the weekend, or something like that, that it doesn’t matter, if I eat healthily most of the time. So, it’s ok to simply treat yourself occasionally*” (Participant 9).

### Experience of prenatal healthcare

This category covered the following subcategories: need for person-centered care, feeling controlled, incoherent healthcare organization, and being lost after labor.

A lack of *person-centered care* was reported. Some felt that they did not get enough information when diagnosed. They also expressed unawareness or ignorance about GDM among healthcare personnel, especially among midwives responsible for the OGTT screening. A few felt stigmatized by healthcare staff, implying that the condition was self-inflicted as previously reported and further felt patronized and questioned during contact with the specialist prenatal care unit.

“*…to be addressed as if you don’t…so…yes as if you knew less than you actually do, or like as if you’re a bit stupid or something, because you ended up in that situation*.” (Participant 10).

One woman expressed that she perceived that the specialist prenatal care unit was understaffed and difficult to get in contact with and that the staff were stressed and under time pressure, which resulted in her feeling like a burden. Some felt that they were left with unanswered questions and lacked support in their fear of future T2DM. This led to a loss of trust and concern about being misunderstood or not taken seriously in future encounters with healthcare. Some expressed that the contact with different endocrinologists by phone felt anonymous and alienating, which made it difficult to provide personal information as one respondent stated:

“*Yes, but you give a lot of information to a doctor. You really have to…especially with the diabetes, it was like they searched every nook and cranny…like, yes what do you eat for breakfast. Like, how do you live…it was important that they knew everything*.” (Participant 1).

Women often felt distressed having to handle the expectations of healthcare personnel. The doctors sometimes gave contradictory advice, which negatively affected trust. On the other hand, many felt safe with and trusted their midwife at their ordinary maternal care unit and would rather not have had to go to the specialist one. A few even sustained additional contact with their usual midwife because of this. Several expressed a positive experience of prenatal care, where they gained enough information and support from doctors, midwives, and dietitians. They thought that reporting their monitored blood glucose by app and receiving a weekly update by phone with a doctor eased the contact with care. Postpartum they also felt supported by the child health services, not regarding GDM but generally in their role as mothers.

Being closely monitored inflicted a sense of being *controlled*. This meant that some felt that the controls were excessive when all turned out to be normal for mother and child.

“*I felt over-cared for, or what to say. That they gave it all, which maybe wasn’t needed*.” (Participant 17).

Hence, a few expressed a wish for individualized care instead, that some women might not need as thorough checkups as stated in the guidelines. In contrast, others felt that the extra level of attention was pleasant and reassuring, especially in relation to their unborn baby’s well-being.

Overall, the women reported uncertainty about how different parts of the *healthcare organization* communicate and collaborate with one another. They expressed a sense of lack of information transfer, forcing the patient herself to be responsible for coordinating her care, which was perceived as challenging.

“*…when I got GDM I was referred to specialist prenatal care, then it felt like they had received a referral with just…’she’s above the threshold’, like that. But there was some other information that I would’ve wished came along, but then I had to tell it instead*.” (Participant 13).

One woman said that the information transfer wasn’t so much lacking as it was non-existent. In general, the participants expressed a wish for a holistic approach concerning their health from the healthcare system, implying that the way healthcare is organized makes this difficult.

”*…the collaboration between primary care and the hospital, it is arranged in silos. And that the transfer isn’t that good…always. Yes, I mean that they are two organizations and it’s like they get so specialized in their silos and they are very capable in their silos, but we patients aren’t arranged in silos, we go across.”* (Participant 13).

Possible solutions suggested were a joint medical record system that both healthcare providers, as well as patients can access, and a couple suggested a kind of “handover meeting” when patients cross from primary to secondary care and vice versa. A few participants reported a trust in information transfer, expressing a belief in the healthcare system. The multidisciplinary prenatal care also caused confusion about who was responsible during which state. This caused uncertainty about where to turn for follow-up both during and after pregnancy. Some felt lost when they tried to get help and perceived that healthcare staff referred to one another, but no one took responsibility, again forcing the patient to coordinate her care.

To be thoroughly (sometimes excessively) followed during pregnancy was in stark contrast with the sense of abandonment many experienced, to be *lost after labor*. In general, women reported that they were informed that they would receive some kind of follow-up sometime after delivery, but many felt unsure by whom and when, perhaps due to the reasons stated above (unclear division of responsibility and lack of information transfer). Being lost after labor also caused increased anxiety about the risk of future T2DM.

## Discussion

### Summary of main findings

This study explored women in the south of Sweden with previous GDM and their experience of diagnosis and GDM care. Most women were initially overwhelmed by the diagnosis and its consequences, both short term as regards the treatment burden, the medicalization of pregnancy, and perceived stigma as well as long-term, with the future risk of T2DM. They felt that healthy eating was important, despite it limiting their everyday life, during pregnancy when the health of their unborn child was of prime concern. Postnatally sustainable eating habits were viewed as the most important. This revealed previous eating disorders and a complex relationship between food and mood, and food as comfort, treating oneself, or as a guilty pleasure. Furthermore, women were well, bordering excessively, taken care of during pregnancy. They were overall satisfied with the provided GDM care but felt abandoned after labor. Women reported uncertainty about healthcare organization, how different healthcare providers collaborate and whose responsibility it was to ensure continuous care. We found that GDM had a major impact on women’s lives and health, labeling them as patients as opposed to expectant mothers.

Women might benefit from being more prepared for the prospective of GDM when conducting the OGTT screening since studies have shown that many women have limited knowledge of the condition beforehand [25, 26]. Sufficient and immediate information about the diagnosis and what to expect from healthcare personnel is crucial to mitigate the initial overwhelming feelings [27]. Healthcare providers need to be aware of how radically the diagnosis affects women’s everyday lives and offer additional support when needed. Likewise, healthcare staff need to be responsive to unhealthy thoughts and behaviors about food and how to address these issues. Perhaps there is a need for increased awareness and knowledge about eating disorders among healthcare personnel in prenatal care. Interestingly, women with GDM rarely mentioned exercise as an alternative to obtain glycemic control during pregnancy and solely seemed to focus on dietary modification, which suggests that there is a need for healthcare personnel to provide more information about physical activity and its blood-glucose lowering effect [28]. Individual care might be difficult to offer since guidelines are general and based on how to manage GDM to minimize the risk of the mother and their offspring. However, each woman has the right to an individual risk assessment and shared decision-making. Women with GDM need healthcare to provide person-centered care and patient-empowerment as opposed to the rather paternalistic approach that many perceived. Healthcare also needs to take responsibility for adequate information transfer to assure continuous care, as well as informing women with GDM of what to expect in terms of long-term follow-up after delivery and ensuring support in how to manage and reduce their future risk of T2DM.

### Comparison with existing literature

Our findings are in line with previous research that highlighted the psychological impact following a GDM diagnosis on women, with the distress of GDM management affecting their mental well-being [20, 21, 29–31]. The initial feelings of shock, fear, uncertainty, and grief have been described in a previous systematic review [22]. This study also reported negative consequences of GDM as added responsibility (eating regimens, appointments), over-medicalization (questioning its necessity), authoritarian healthcare personnel and a lack of individualized care as well as the experience of intensive intervention and then nothing after delivery. One described benefit of GDM in this study and others was the opportunity to improve health and make healthy eating changes [22, 32]. Craig et al. in contrast to the current study, found a low awareness of GDM long-term risks as another key finding [22]. Women’s risk perception of future T2DM might be influenced by in which context they are being followed during pregnancy, as one Swedish study found. Women monitored at a diabetes specialist clinic expressed fear of future T2DM whereas women monitored at a specialist maternity clinic believed the condition to be transient [27]. Parsons et al. also revealed overwhelming feelings, among women with GDM in the UK, described as “the disrupted pregnancy” [21]. Furthermore, they reported the feeling of being over-scrutinized as “projected anxiety” from healthcare providers prioritizing the health of the baby and the perceived stigma, being labeled as “diabetic” rather than “pregnant” as well as the feeling of postpartum abandonment. Suggested improvements in GDM care were increased emotional support, reduced medicalization, a more motivational approach, and enhanced postpartum care [21]. The feelings of uncertainty, disbelief, and skepticism about diagnosis that some women reported in our study has not been previously well documented, but we found it described in one other study [32]. These feelings might also be associated with the attached stigma, not wanting to identify with the diagnosis [33]. Despite the initial overwhelming feelings, women gradually adapted to the diagnosis, found acceptance and balance, and gained control, as previously reported [18, 20, 22].

Earlier research has also described the stigma associated with GDM by healthcare personnel as discrimination but also internalized as feelings of failure, guilt, shame, and self-blame, which also were evident in the present study [20, 29]. Identified potential adverse consequences of stigma such as not following dietary advice or reporting monitored blood glucose, avoiding screening, social isolation, and poor mental health, have been reported [20, 29, 33, 34]. Social and familial support as well as self-management and healthcare staff attending to women’s physiological health has been highlighted as coping strategies to reduce stigma [29]. The medicalization of pregnancy is not just an issue with GDM, but in general as healthcare focuses on risk reduction, implementing controlling activities affecting pregnant women’s personal autonomy and neglecting their need for comprehensive care [21, 35]. Others have even suggested that the current biomedical model in GDM management is ‘fetal-centric’ and fails to acknowledge the well-being of diagnosed women [31, 35].

This study provided many interesting views on diet, both in relation to managing GDM as well as reducing future T2DM risk. Although little is known about disordered eating behaviors among women with GDM, a previous study also described that GDM disrupted women’s relationship to food and could trigger “medically promoted” eating disorders [36]. The systematic review previously referred to also reported extreme behaviors such as purging and starvation, as well as Draffin et al. [22, 25]. On the other hand, an additional study found that women with GDM thought that the dietary advice from dietitians was perceived as manageable and as an opportunity to enact dietary changes. This study also suggested that changes in physical activity were perceived as more challenging and associated with greater stress [37]. However, when dietary modifications are done mainly to maintain blood glucose levels within limits and for the sake of the expected child, and are not perceived as optimizing health, they might be more difficult to sustain in the long-term, as one study has suggested [38].

In a Norwegian qualitative study, they found a need for improved collaboration between secondary and primary care to avoid women having to coordinate their own care [19]. The division of GDM surveillance between different healthcare providers can also contribute to a lack of comprehensive approach that women perceive about their GDM care as reported by others as well as in the present study [39, 40]. The necessity of improved transitions was reflected in a systematic review that also claimed that discontinuity between hospital-based and primary healthcare services results in missed opportunities to assist women with GDM in sustaining healthy lifestyles to prevent future disease. This review also emphasized the lack of individualized care and women’s need for a holistic, person-centered approach from healthcare staff to improve their GDM experience as well as long-term follow-up in primary care [35]. A previous study that we conducted in southern Sweden demonstrated a lack of follow-up and screening for T2DM in women with previous GDM in primary care, suggesting inadequate communication between secondary and primary care as one possible explanation [41].

### Strengths and limitations

This qualitative study has several strengths that contribute to its trustworthiness. First, we managed to recruit participants of different ages, parity, socioeconomic status, habitation, and ethnicity to achieve credibility and increase transferability. Credibility was also facilitated by investigator triangulation during analysis as well as recurrent summaries during the interviews as part of member checks. The first author conducting the interviews also presented preliminary findings to midwives outside of the study at the midwifery clinic. Second, one of the senior authors first got involved with the study in a later stage during data analysis, thus increasing the dependability. The use of a semi-structured interview guide, as well as variation in sample, further contributes to this. Finally, all participants gave permission to later contact if further information was needed as part of participant evaluation, although this wasn’t required. Evidently, this study also has its limitations. All involved researchers work in primary care, which affects our preunderstanding. However, the first author also works with pregnant women at a midwifery clinic, collaborating with clinicians in obstetrics/endocrinology and has also visited the specialist prenatal care unit as part of doing research before this study was conducted. Another limitation that could affect the trustworthiness is the lack of patient public involvement in designing the study, except for the pilot interviews. All participants were interviewed a couple of months to years after their diagnosis, which of course can contribute to recall bias. There is also the possibility of selection bias since women with less positive experiences of the care provided might have been more likely to participate. As for all qualitative interview studies, there is also the risk of social desirability bias since the results are based on self-reported experiences, which perhaps also may be influenced by the interviewer being a doctor. The interviews and analysis were conducted in Swedish, with the results thoroughly translated to English, but there is always the small risk of content being lost in translation. Finally, the results are not generalizable but can only be applied to an utterly similar context.

### Implications for research and practice

There is a very limited number of qualitative studies conducted in Sweden exploring GDM women, and there certainly have not been any recent ones, until now [18, 27, 40]. This study provides a better understanding of how Swedish women with GDM experience the diagnosis and prenatal healthcare. GDM has wide-reaching consequences on women’s lives as well as pregnancies, suddenly becoming a patient as opposed to an expectant mother. We also found it interesting that food seems to be a sensitive topic, both during and after pregnancy. On the other hand, that might be expected when diet is such a central part of managing GDM and preventing T2DM. But perhaps healthcare personnel need to think about how they convey this message. How women with GDM perceive prenatal care might also influence their attitudes toward future care, having to live with the increased risk of T2DM and to which extent they adopt preventive lifestyle behaviors. We have the potential to improve care by increasing the awareness among clinicians about this patient group experience. It is also evident that the transition between secondary and primary care needs to be improved to bridge the abandonment after labor and secure lifelong follow-up and screening for T2DM. Future studies need to explore women with a history of GDM and how they experience follow-up in primary care and lifestyle interventions to identify approaches to prevent T2DM, which we are planning.

## Conclusions

This qualitative study of women with a history of GDM in southern Sweden found that GDM has a major impact on women’s lives and pregnancies, suddenly becoming a patient as opposed to an expecting mother. Future GDM care can be improved by additional information and emotional support by healthcare personnel, increased awareness and understanding of the treatment burden of GDM especially considering dietary modifications, improved person-centered care, as well as better collaboration between healthcare providers, particularly to ensure comprehensive post-pregnancy follow-up.

## Data Availability

According to Swedish legislation about ethical permission and data protection, restrictions apply to the availability of these data, which were used under license for the current study. However, the datasets used and analyzed during the current study are available from the corresponding author upon reasonable request.

## Abbreviations

GDM: Gestational diabetes mellitus
T2DM: Type 2 diabetes mellitus
OGTT: Oral glucose tolerance test
BMI: Body mass index
